# 

**Published:** 1914-12

**Authors:** 


OFFICIAL ORGAN—State Society Social Hygiene and Eugenics.

Vol. XXX DECEMBER, 1914 No. 6

THIRTIETH YEAR

### The "Red Back"

### TEXAS MEDICAL JOURNAL

ESTABLISHED JULY, 1885

PUBLISHED MONTHLY AT AUSTIN, TEXAS, BY

MRS. F. E. DANIEL, - - - Publisher and Managing Editor

PRINTED BY THE VON BOECKMANN-JONES CO.

Subscription, $1.00 a Year, in Advance. - - - - Single Copies, 10 Cents

Entered at the Postoffice at Austin, Texas, as second class mail matter.

